# *Mycobacterium chelonae* as an Underrecognized Cause of Granulomatous Disease in Fish and Humans: A Molecular One Health Study

**DOI:** 10.3390/pathogens15080856

**Published:** 2026-08-17

**Authors:** Tiziana Cubeddu, Luca Pilloni, Giovanni Pietro Burrai, Marina Antonella Sanna, Marta Polinas, Claudio Murgia, Clara Gerosa, Rossano Ambu, Daniela Fanni, Elisabetta Antuofermo

**Affiliations:** 1 Department of Veterinary Medicine, University of Sassari, 07100 Sassari, Italy; tcubeddu@uniss.it (T.C.); masanna1@uniss.it (M.A.S.); mpolinas@uniss.it (M.P.); claudiomurgia04@gmail.com (C.M.); eantuofermo@uniss.it (E.A.); 2 Division of Pathology, Department of Medical Sciences and Public Health, University Hospital of Cagliari, 09124 Cagliari, Italy; lucpilloni@tiscali.it (L.P.); clara.gerosa@unica.it (C.G.); amburo@unica.it (R.A.); daniela.fanni@unica.it (D.F.); 3 Mediterranean Center for Disease Control, University of Sassari, 07100 Sassari, Italy

**Keywords:** non-tuberculous mycobacteria, *M. chelonae*, granulomatous dermatitis, fish mycobacteriosis, Ziehl–Neelsen staining, molecular diagnostics, One Health

## Abstract

Non-tuberculous mycobacteria are recognized as pathogens affecting aquatic animals and humans. Although *Mycobacterium marinum* is traditionally considered the principal etiological agent of fish mycobacteriosis and fish-tank granuloma in humans, the contribution of *Mycobacterium chelonae* in fish as well as in humans remains underestimated, particularly in Ziehl–Neelsen (ZN)-negative lesions. In this study, granulomatous lesions of marine fish species and human skin biopsies were investigated using histopathology and molecular diagnostics. Following histological evaluation, all samples were analysed by PCR amplification and sequencing of the *hsp65* gene to identify the causative agent. Fish and human tissues showed similar chronic granulomatous lesions, with all cases negative for acid-fast bacilli by ZN staining. Molecular analysis consistently identified *M. chelonae* in both marine fish organs and human skin lesions exhibiting clinicopathological features traditionally attributed to *M. marinum*. Phylogenetic analysis revealed high genetic similarity between fish- and human-derived sequences, suggesting shared environmental reservoirs rather than host-specific lineages. These findings identify *M. chelonae* as an underrecognized cause of granulomatous disease in fish and humans and demonstrate the limitations of histochemical methods in these infections. Incorporating molecular diagnostics into routine investigations of granulomatous disease supports more accurate diagnosis across veterinary and human medicine and strengthens One Health surveillance of aquatic non-tuberculous mycobacterial infections.

## 1. Introduction

Nontuberculous mycobacteria (NTM) are ubiquitous environmental bacteria inhabiting soil and aquatic ecosystems. Thanks to their remarkable ecological plasticity, NTM persist in both natural and engineered habitats, such as plumbing systems and biofilms [1]. Given their ability to cause opportunistic infections in humans and animals, understanding their distribution and transmission routes is a growing priority. Among aquatic species, NTM cause fish mycobacteriosis—a chronic, granulomatous disease that inflicts severe losses on aquaculture and poses a One Health zoonotic risk [2,3].

Several NTM species have been implicated in fish mycobacteriosis, including *Mycobacterium marinum*, *M. chelonae*, *M. fortuitum*, *M. shottsii*, *M. pseudoshottsii*, *M. ulcerans*, *M. abscessus*, *M. salmoniphilum*, *M. haemophilum*, and *M. gordonae* [1,2]. Among rapidly growing NTM, *M. salmoniphilum*, a species closely related to the *M. chelonae–abscessus* group, has also been associated with systemic granulomatous disease in farmed Russian sturgeon [4]. In the Mediterranean area, *M. marinum* has been confirmed as the cause of systemic mycobacteriosis in reared mullets from Sardinia, demonstrating the circulation of zoonotic NTM in fish populations from the same geographical region investigated in the present study [5]. Among these, *M. marinum* and *M. chelonae* are of particular interest because they infect both fish and humans [6,7,8]. While *M. marinum* has historically been regarded as the principal fish-associated zoonotic mycobacterium, increasing evidence suggests that *M. chelonae* represents an underrecognized pathogen whose epidemiological and clinical relevance has likely been underestimated.

*Mycobacterium chelonae*, a fast-growing species of the *M. chelonae–abscessus* complex, persists in aquatic environments and sediments through biofilm formation. Environmental stress (e.g., poor water quality, high temperatures, overcrowding) promotes its transmission. Following entry through damaged skin, gills, or ingestion, the bacterium can survive within macrophages and induce chronic granulomatous inflammation, facilitating long-term infection [2].

*M. chelonae* is a recognised cause of fish mycobacteriosis, a chronic systemic disease characterised by multifocal granulomatous inflammation affecting multiple visceral organs. The infection has been reported in numerous freshwater and marine fish species, including gilthead seabream (*Sparus aurata*), European seabass (*Dicentrarchus labrax*), mullets (Mugilidae), and, more recently, meagre (*Argyrosomus regius*), in which extensive visceral granulomas have been described [9,10]. In Russian sturgeon (*Acipenser gueldenstaedtii*), *M. chelonae* has also been molecularly identified in chronic granulomatous skin and oral lesions, despite acid-fast bacilli being detected by Ziehl–Neelsen staining in only one lesion [11].

In humans, extrapulmonary NTM infections predominantly involve skin and soft tissues and are increasingly recognised following trauma, cosmetic procedures, or exposure to contaminated aquatic environments [12]. Fish-associated granulomatous dermatitis—historically referred to as fish tank granuloma—has been almost exclusively attributed to *M. marinum* and typically develops after minor skin injuries from contact with aquaria, infected fish, or contaminated water [13,14,15,16]. However, accumulating clinical evidence indicates that *M. chelonae* can produce remarkably similar cutaneous manifestations, making clinical and histopathological differentiation from *M. marinum* particularly challenging [17,18]. Despite these observations, the role of *M. chelonae* as a causative agent of fish-associated granulomatous dermatitis remains poorly defined and is likely underestimated.

A common pathological hallmark of NTM infection in both fish and humans is the development of chronic granulomatous inflammation, reflecting conserved host immune responses against persistent intracellular pathogens. In fish, granulomas typically consist of concentric aggregates of epithelioid macrophages, lymphocytes, and central necrotic areas in visceral organs [19,20], whereas human cutaneous lesions frequently exhibit diffuse or multifocal granulomatous dermatitis with variable inflammatory patterns depending on the host immune status [21]. However, accurate diagnosis of NTM infections remains challenging. Ziehl–Neelsen (ZN) staining is routinely employed to detect acid-fast bacilli within granulomatous lesions; however, false-negative results are common in paucibacillary infections, in degraded formalin-fixed tissues, or when bacilli are obscured by extensive tissue necrosis [17,22]. Although the Fite–Faraco stain may improve bacillary detection in formalin-fixed paraffin-embedded (FFPE) tissues, its diagnostic sensitivity remains substantially lower than that of molecular assays [23,24]. Furthermore, culture-based identification is often limited by prolonged incubation times, contamination, and the fastidious growth characteristics of several NTM species [25].

Consequently, molecular diagnostics have become an essential component of contemporary pathology. PCR amplification followed by sequencing of conserved genetic targets, including *hsp65*, *rpoB*, and 16S rRNA, enables rapid and accurate species identification even from FFPE tissues, overcoming many of the limitations associated with conventional histopathological and microbiological methods [10,26,27,28,29].

Against this background, the present study investigated granulomatous lesions from FFPE tissues of marine fish and human skin biopsies using an integrated histopathological and molecular approach. By combining comparative pathology with *hsp65*-based molecular identification and phylogenetic analysis, we aimed to evaluate the role of *Mycobacterium chelonae* as an underrecognized cause of granulomatous disease across fish and humans. We further sought to demonstrate the diagnostic value of molecular analysis in Ziehl–Neelsen-negative granulomatous lesions and to provide new evidence supporting a One Health perspective for the diagnosis and surveillance of fish-associated non-tuberculous mycobacterial infections.

## 2. Materials and Methods

### 2.1. Sample Collection

A total of 30 formalin-fixed, paraffin-embedded (FFPE) archival samples from Ziehl–Neelsen (ZN)-negative granulomatous lesions were retrospectively selected from 26 fish, including 13 *Mugilidae*, 9 *Sparus aurata*, and 4 *Dicentrarchus labrax*. Specifically, 15 samples from Mugilidae (11 livers, 2 spleens, 1 brain, and 1 gonad), 11 specimens from *Sparus aurata* (3 livers, 3 spleens, 3 hearts, 1 kidney, and 1 gonad), and 4 organs from *Dicentrarchus labrax* (1 kidney, 1 heart, 1 spleen, and 1 liver), for a total of 30 samples, were analysed.

All fish samples originated from archival material and were collected for diagnostic purposes. Fish necropsies were performed at the Department of Veterinary Medicine, University of Sassari, Italy. Tissue aliquots were fixed in 10% neutral buffered formalin for 48 h for histopathological examination. In addition, 12 FFPE human skin biopsies collected for routine diagnostic purposes were retrieved from the Division of Pathology, Department of Medical Sciences and Public Health, University Hospital of Cagliari (Italy). Histopathological diagnoses included nodular (*n* = 7), diffuse (*n* = 2), and interstitial (*n* = 3) granulomatous dermatitis, with variable mixed inflammatory infiltrates predominantly composed of lymphocytes; one biopsy was diagnosed as suppurative dermatitis.

### 2.2. Histopathology

A retrospective cohort of archival formalin-fixed tissue samples from both fish and human sources was assembled for this analysis. Samples were dehydrated through graded alcohols and xylene and embedded in paraffin (FFPE), using an automatic tissue processor. Serial sections 3 µm thick were cut using a microtome (RM2245, Leica Biosystems, Wetzlar, Germany) and stained with hematoxylin and eosin (H&E) using an automatic multistainer (ST5020, Leica Biosystems, Wetzlar, Germany). As part of the routine diagnostic workflow, sections from fish organs and human skin biopsies displaying granulomatous inflammation were additionally stained using the Ziehl–Neelsen (ZN) method for the detection of acid-fast bacilli, following a previously described standard protocol [10]. Briefly, sections were deparaffinized, treated with periodic acid for 10 min at room temperature and stained with carbolfuchsin for 30 min at room temperature. Sections were then rinsed under running cold tap water for 2 min, differentiated in acid alcohol (95% ethanol containing hydrochloric acid), and then briefly rinsed in distilled water. Slides were then counterstained with methylene blue, washed in distilled water, dehydrated, cleared and mounted with a coverslip and a permanent medium.

Histological lesions were systematically evaluated and categorized according to the type of inflammatory response, distribution, and severity. Human skin biopsies were independently assessed by two medical pathologists (MD), while fish tissues were evaluated by two veterinary pathologists (DVM). Representative photomicrographs were acquired using a Nikon Digital Sight DS-U1 camera mounted on a Nikon Eclipse 80-i microscope (Nikon Corporation, Kanagawa, Japan).

### 2.3. Molecular Identification

#### 2.3.1. DNA Extraction

DNA extraction was performed from FFPE samples of fish organs and human skin biopsies. For each FFPE block, fifteen serial sections (3 μm thick) were collected into sterile 1.5 mL microcentrifuge tubes. To minimize cross-contamination, microtome blades were replaced between samples, and all instruments and working surfaces were thoroughly decontaminated before processing each block.

Tissue sections were deparaffinized using xylene. Briefly, the sections were immersed in 1 mL of xylene, vortexed, and centrifuged at 10,000× *g* for 10 min. The supernatant was carefully removed, and the pellet was washed twice with 100% ethanol to eliminate residual xylene, followed by centrifugation at 14,000× *g* for 5 min. The resulting pellets were air-dried at 37 °C for 15–20 min to evaporate any remaining ethanol.

DNA was then extracted using the DNeasy Blood and Tissue Kit (Qiagen, Hilden, Germany) according to the manufacturer’s protocol. DNA concentration and purity were measured using a NanoDrop ND-2000 spectrophotometer (Thermo Fisher Scientific, Waltham, MA, USA).

#### 2.3.2. Polymerase Chain Reaction (PCR)

Molecular identification and characterisation of *Mycobacterium* spp. were performed by PCR amplification and sequencing of the 65-kDa heat shock protein (*hsp65*) gene on FFPE samples. To exclude other bacterial pathogens potentially associated with granulomatous lesions, additional PCR assays targeting *Nocardia* spp., *Photobacterium damselae* subsp. *damselae*, and *Photobacterium damselae* subsp. *piscicida* were also performed.

Partial amplification of the *hsp65* gene (~441 bp) was carried out according to Telenti et al. [26], slightly modified to amplify only the mycobacterial *hsp65* from tissue. The PCR mix contained 1 × reaction buffer (with 1.5 mM MgCl2), 1 × CoralLoad, 200 mM dNTP, 25 pmol of each primer, 1U Taq DNA polymerase (Qiagen), and 5 µL of total DNA in 50 µL of reaction. PCR amplification consisted of an initial denaturation at 93 °C for 10 min, followed by 45 cycles of 94 °C for 1 min, 61 °C for 40 s, and 72 °C for 1 min, with a final extension at 72 °C for 7 min. PCR products were analysed by 2% agarose gel electrophoresis, and amplicons were detected using GelRed staining and a UV transilluminator. *Mycobacterium marinum* (DSM 44344—ATCC 927) and *Mycobacterium fortuitum* (DSMZ 46621—ATCC 6841) were used as positive controls. DNA isolated from the spleen and liver of healthy fish without granulomas and nuclease-free water were used as negative controls.

The amplification of a 16S ribosomal RNA (rRNA) gene segment specific to the genus *Nocardia* was performed using NG1 and NG2 primers [30]. The amplification program was as follows: 10 min for the initial denaturation step at 94 °C, 30 cycles (94 °C for 60 s, 52 °C for 20 s, 72 °C for 60 s) and a 10 min final extension step at 72 °C. Amplification was evaluated by means of 1.5% agarose gel electrophoresis, and the presence of a 596 bp fragment was regarded as a positive result.

The PCR for *Photobacterium damselae* subspecies *piscicida* was conducted on the gene coding for a putative penicillin-binding protein (1A), using PdP forward and Pdp reverse primers that amplify a 297 bp fragment [31]. On the other hand, primers (forward UreC and reverse UreC) for subspecies *damselae* amplify a 448 bp fragment of the enzyme UreasiC gene [32]. Amplification was carried out using the following cycling program: initial denaturation step at 95 °C for 4 min, 50 cycles of denaturation at 95 °C for 30 s, primer annealing at 65 °C for 30 s, extension at 72 °C for 1 min, and a final extension at 72 °C for 10 min.

Negative and positive controls (DNA of *Photobacterium damselae* subsp. *damselae* ATCC 33539 and *Photobacterium damselae* subsp. *piscicida* ATCC 29688) were included in each amplification series.

The amplicons of ~440 bp in size were gel-purified using the QIAquick Gel Extraction Kit (Qiagen) and subjected to Sanger sequencing in service at the BMR-genomics Srl. The electropherograms were manually edited for sequence accuracy using BioEdit v. 7.2.5 [33]. Subsequently, the sequences were queried against GenBank’s Basic Local Alignment and Search Tool (BLAST version 2.17.0) at https://www.ncbi.nlm.nih.gov/ (accessed on 2 March 2026) to identify closely related sequences. For phylogenetic analysis, MEGA v.12 software was employed [34]. The phylogenetic tree was constructed using the maximum-likelihood method, and evolutionary distances were calculated by the Kimura 2-parameter model with 1000 bootstrap replicates.

## 3. Results

### 3.1. Histopathology

**Figure 1:**
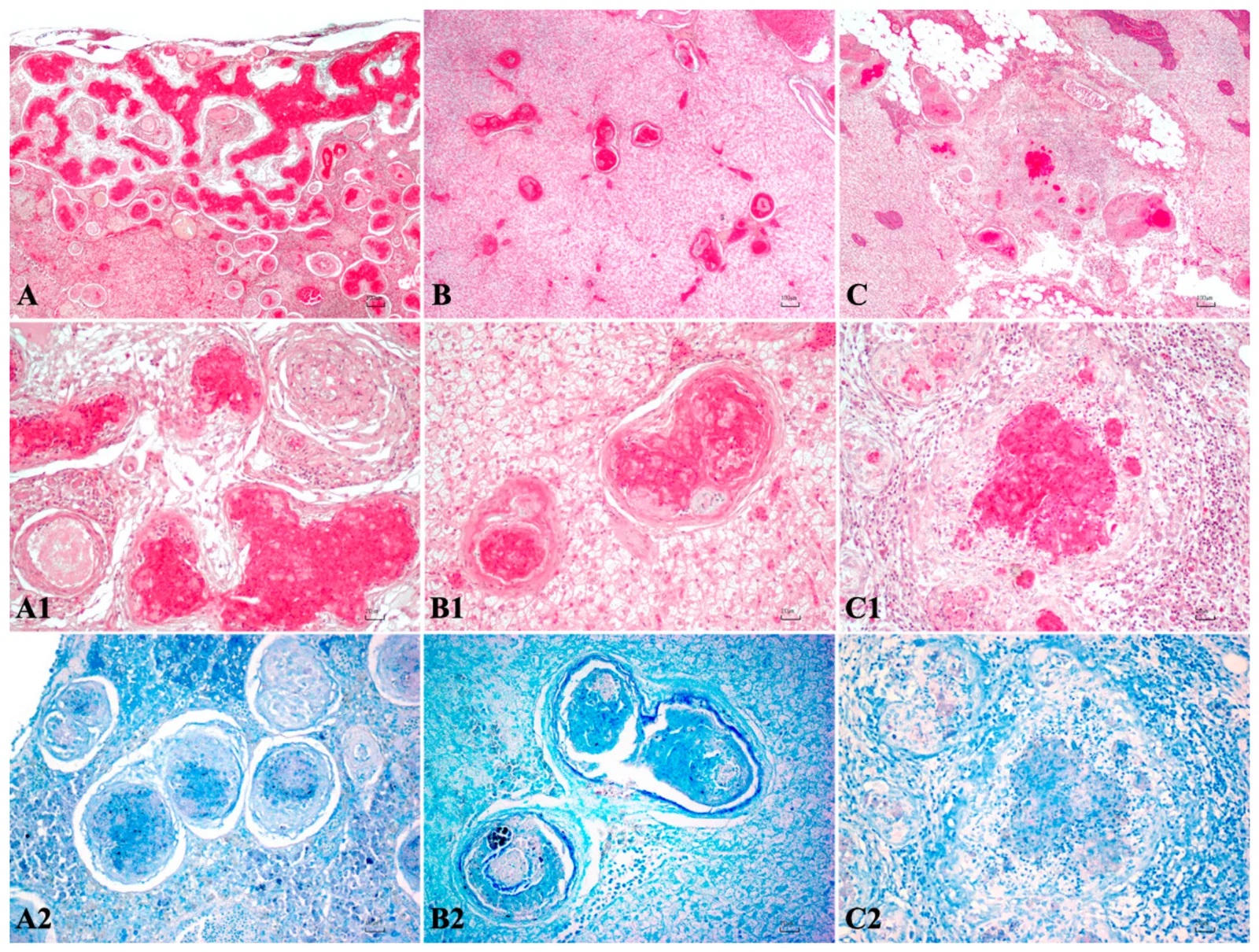
Representative histopathological features of granulomatous lesions in fish tissues (**A**–**C2**). (**A**–**A2**) Mugilidae liver showing severe multifocal to coalescing granulomatous hepatitis with central necrosis surrounded by epithelioid macrophages. (**B**–**B2**) *Dicentrarchus labrax* liver with multifocal granulomatous hepatitis characterized by a necrotic core surrounded by epithelioid macrophages and spindle cells. (**C**–**C2**) *Sparus aurata* liver displaying multifocal granulomatous hepatitis with abundant macrophages and lymphocytic infiltrates. Ziehl–Neelsen staining was negative for acid-fast bacilli in all lesions. H&E; scale bars: 100 μm (**A**–**C**) and 20 μm (**A1**–**C2**).

Histopathological evaluation of fish tissues consistently revealed granulomatous lesions affecting all examined organs. Lesions were predominantly multifocal with a moderate to severe degree of inflammatory reaction. Individual granulomas ranged from 200 to 900 μm in diameter, consisting of a central eosinophilic necrotic core containing granular debris, surrounded by concentric layers of epithelioid macrophages intermixed with lymphoplasmacytic inflammatory cells. An outer rim of elongated spindle-shaped cells, consistent with fibroblasts, was frequently observed, whereas multinucleated giant cells were absent in all cases. Despite the well-developed granulomatous architecture, Ziehl–Neelsen staining was negative in all examined samples, with no acid-fast bacilli detected (Figure 1).

**Figure 2:**
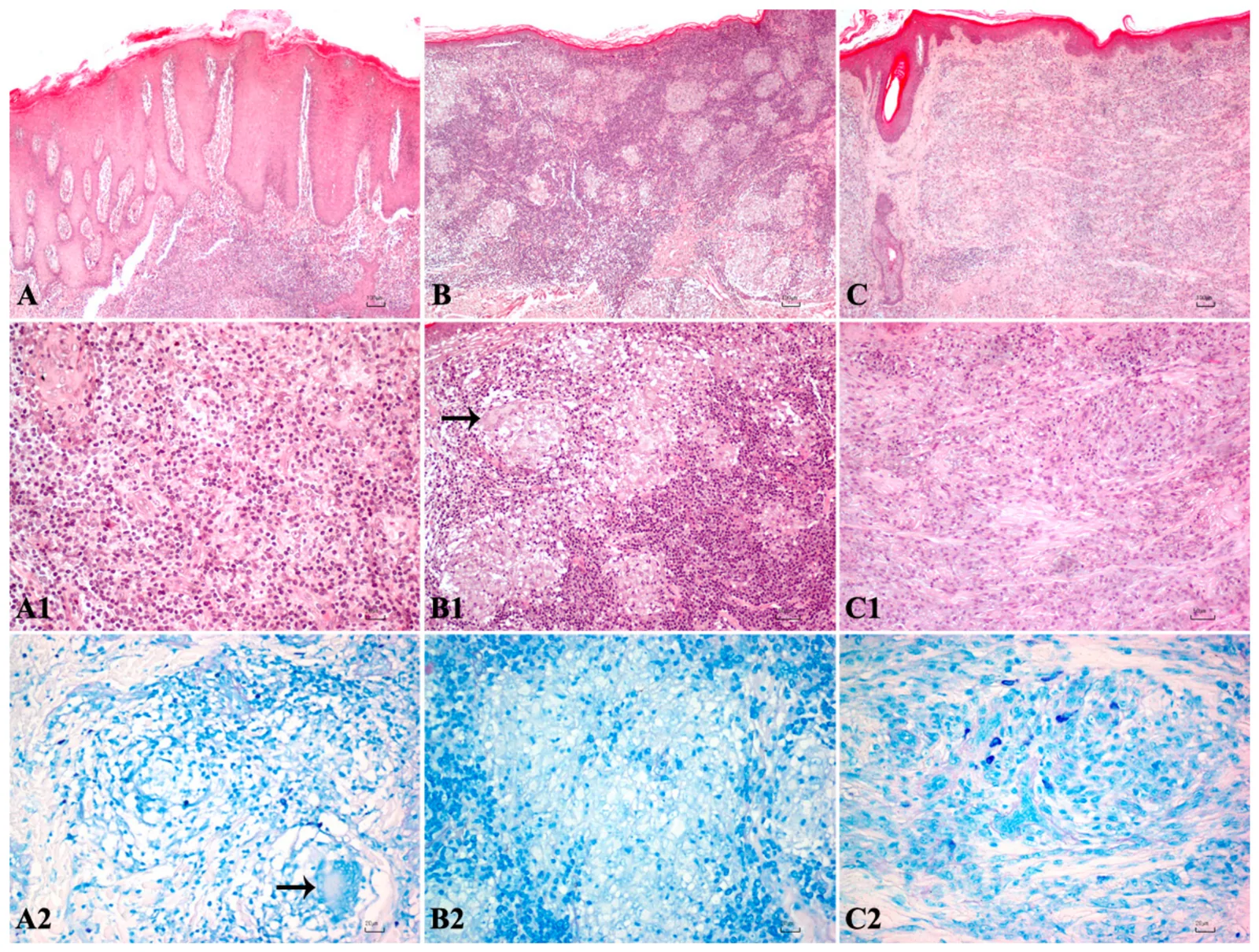
Representative histopathological features of human granulomatous dermatitis (**A**–**C2**). (**A**–**A2**) Diffuse granulomatous dermatitis with lymphohistiocytic infiltrates extending throughout the dermis with scattered multinucleated giant cells (arrow). (**B**–**B2**) Nodular granulomatous dermatitis composed of epithelioid histiocytes with scattered multinucleated giant cells (arrow). (**C**–**C2**) Interstitial non-caseating granulomatous dermatitis with clustered histiocytes embedded within fibrous connective tissue. Ziehl–Neelsen staining was negative for acid-fast bacilli in all cases. H&E; scale bars: 100 μm (**A**–**C**) and 20 μm (**A1**–**C2**).

In human skin samples, histopathological examination revealed a granulomatous dermatitis extending from the superficial to the middle and deep dermis. The inflammatory infiltrate was predominantly composed of macrophages mixed with lymphocytes and plasma cells, arranged in nodular (7/12), diffuse (2/12), or interstitial (3/12) patterns. Histiocytes were readily identifiable by their abundant eosinophilic and frequently vacuolated cytoplasm, ovoid to reniform nuclei with variably prominent nucleoli. Multinucleated giant cells were evident in some cases (Figure 2). All examined skin biopsies tested negative for acid-fast bacilli by Ziehl–Neelsen staining.

### 3.2. Molecular Detection and Sequencing of Mycobacterium spp.

PCR amplification of the *hsp65* gene in fish specimens yielded an expected ~440 bp amplicon in 20 out of 30 samples (66.7%). Positivity was detected in 9/15 (60.0%) Mugilidae tissues, 7/11 (63.6%) *S. aurata* tissues, and all four *D. labrax* samples (100%). Positive samples were most frequently obtained from the liver (11/20), followed by the spleen (4/20), heart (3/20), kidney (1/20), and gonad (1/20). All fish samples tested negative for *Photobacterium damselae* subsp. *damselae*, *P. damselae* subsp. *piscicida* and Nocardia spp. Results of molecular investigation on fish samples are summarised in Table 1.

PCR amplification of the *hsp65* gene was positive in 6 out of 12 (50%) human skin biopsies, including 5 out of 6 samples from the upper limbs and 1 out of 6 samples from the lower limbs. All samples tested negative for *Photobacterium damselae* subsp. *damselae*, *Photobacterium damselae* subsp. *piscicida*, and Nocardia spp. Molecular results for human biopsies are reported in Table 2.

Analysis of PCR-positive sequences of *hsp65* identified mycobacteria in 20 out of 20 (100%) fish and in 6 out of 6 human samples. The obtained sequences shared 99–100% nucleotide identity with *Mycobacterium chelonae* strains available in the GenBank database. Regarding human samples, positivity for *Mycobacterium chelonae* was almost exclusively localized to the extremities.

**Figure 3:**
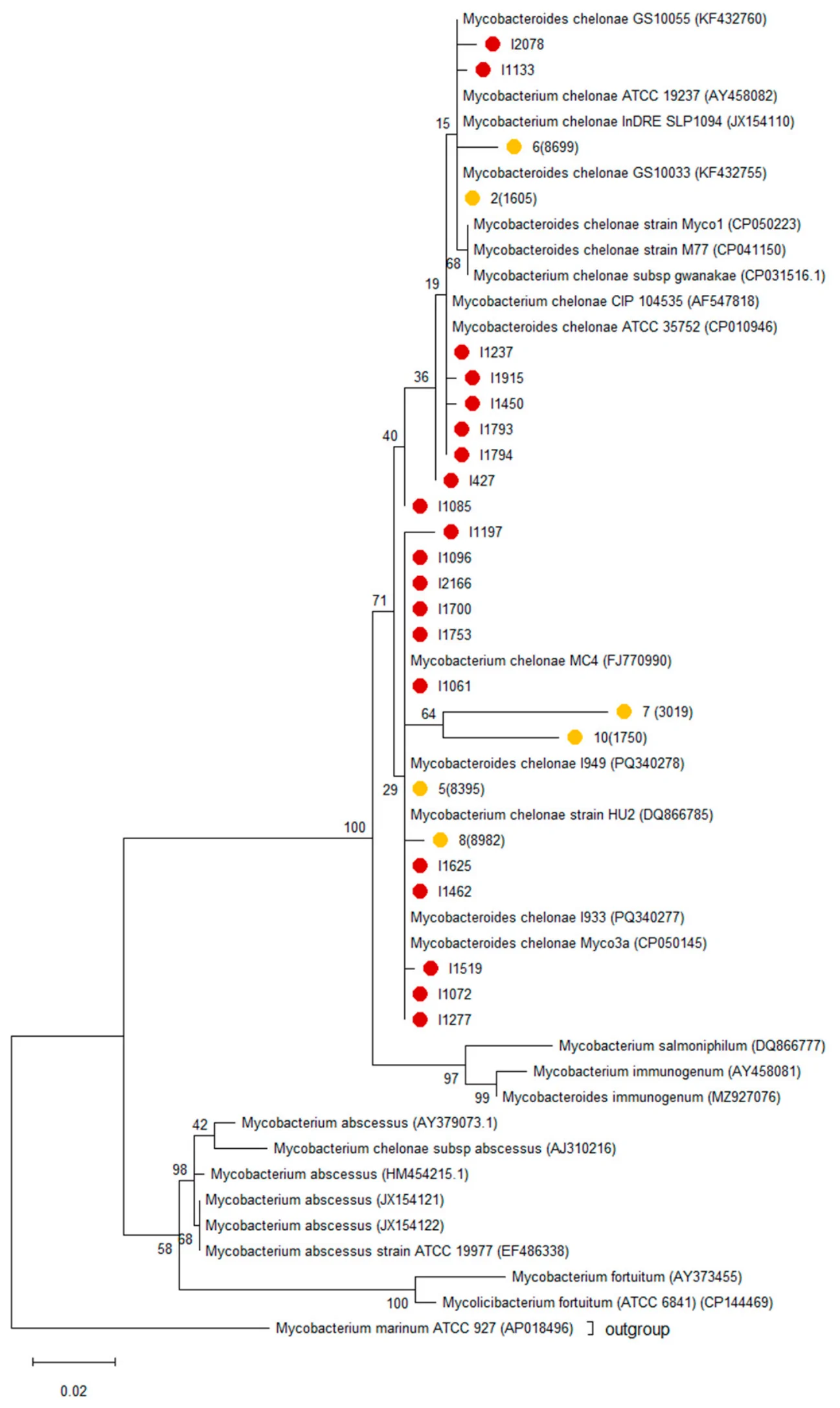
Phylogenetic tree based on partial sequence of the *hsp65* gene. The tree was constructed by the ML method with the Kimura-2p + G model and 1000 bootstrap replicates. Sequences obtained from fish samples are indicated by red dots, whereas sequences from human biopsies are indicated by yellow dots. *Mycobacterium marinum* was used as the outgroup.

Sequences were aligned using Clustal W software (version 2.1), and the ML phylogenetic tree was built by MEGA12 software. Maximum-likelihood phylogenetic analysis based on partial *hsp65* sequences showed that isolates obtained from fish and human samples clustered together within the *M. chelonae* clade, without evident host-specific segregation (Figure 3). *Mycobacterium marinum* was included as the outgroup. The GenBank accession numbers of the *hsp65* sequences obtained in this study, along with the corresponding sample IDs, are reported in Table 3.

## 4. Discussion

The present study demonstrates that molecular analysis of formalin-fixed, paraffin-embedded (FFPE) tissues substantially improves the detection of non-tuberculous mycobacteria (NTM) in chronic granulomatous lesions that remain negative by Ziehl–Neelsen (ZN) staining. By combining histopathology with PCR amplification and sequencing of the *hsp65* gene, *Mycobacterium chelonae* was consistently identified in both marine fish and human skin biopsies, supporting its role in chronic granulomatous disease and highlighting the diagnostic limitations of conventional histochemical methods in paucibacillary infections.

Histopathological examination of fish tissues revealed well-organized granulomas characterized by central eosinophilic necrosis surrounded by epithelioid macrophages and peripheral spindle cells, consistent with the classical description of piscine mycobacteriosis [3,7]. Similar lesions have previously been described in infections caused by both *M. marinum* and *M. chelonae* in several teleost species [5,6,11,35]. Despite these characteristic morphological features, acid-fast bacilli were not detected in any sample by ZN staining. This finding is consistent with previous observations in both veterinary and human pathology, where mycobacterial infections may remain undetected because of the low bacterial burden or reduced acid-fastness following formalin fixation and paraffin embedding [36,37]. Consequently, negative ZN staining should not be considered sufficient to exclude mycobacterial infection when chronic granulomatous inflammation is present.

Molecular analysis overcame this diagnostic limitation by enabling species-level identification directly from FFPE tissues. Sequencing of the *hsp65* amplicons identified *M. chelonae* in all PCR-positive samples, confirming the suitability of this genetic marker for the identification of rapidly growing mycobacteria, as previously demonstrated [26,38]. The successful amplification of *hsp65* from archival FFPE material further demonstrates the applicability of molecular diagnostics in retrospective investigations.

The predominance of *M. chelonae* observed in the present study is noteworthy because piscine mycobacteriosis has historically been associated primarily with *M. marinum* and *M. fortuitum*, although *M. salmoniphilum* has also been reported in cultured fish [4,7]. Increasing reports over the last decade, however, indicate that rapidly growing mycobacteria, particularly *M. chelonae*, are emerging pathogens in both aquaculture and human medicine [11,24]. Moreover, in a study conducted on environmental nontuberculous mycobacterial diversity by Gebert and colleagues (2026), clinically relevant NTM were mainly associated with household plumbing biofilms, where *M. mucogenicum/phocaicum*, *M. avium*, *M. abscessus*, and *M. chelonae* complexes showed the highest occurrence. Notably, the presence of the *Mycobacterium chelonae* complex in surface waters indicates its persistence in the environment, suggesting that aquatic ecosystems can be a major source of infection by this opportunistic pathogen [1]. Nevertheless, this data aligns with our findings. The widespread detection of *M. chelonae* in the present series suggests that its role in granulomatous disease may have been previously underestimated, also in human samples. Furthermore, recent ecological studies underscore the growing recognition of NTM, including *M. chelonae,* as persistent, climate-sensitive opportunistic pathogens, indicating that changing environmental factors can shape their distribution, persistence, and clinical emergence [39].

The high nucleotide identity observed between fish and human isolates supports the hypothesis of common environmental reservoirs rather than host-specific lineages. *Mycobacterium chelonae* is widely distributed in natural and artificial aquatic environments, including biofilms and water distribution systems, where its intrinsic resistance to adverse environmental conditions promotes long-term persistence [17]. Although the present study does not demonstrate direct zoonotic transmission, the identification of genetically closely related strains in aquatic animals and humans reinforces the importance of environmental exposure within a One Health context.

The remarkable histopathological similarities observed between piscine and human granulomas further strengthen this concept. In both hosts, granulomas consisted predominantly of epithelioid macrophages organized around a necrotic core and accompanied by chronic inflammatory infiltrates, reflecting highly conserved host responses to intracellular mycobacteria. Granuloma formation represents a dynamic balance between bacterial containment and persistence, providing a microenvironment in which mycobacteria may evade complete immune clearance while limiting tissue dissemination [40,41].

Traditionally, fish tank granuloma has been almost exclusively attributed to *Mycobacterium marinum*. The present findings, together with accumulating evidence from the literature, suggest that *M. chelonae* should likewise be recognized as a causative agent of this clinical entity. Because the lesions produced by the two species are histopathologically indistinguishable, species identification relies on molecular methods rather than morphology alone.

These similarities support the use of naturally occurring piscine mycobacteriosis as a comparative model for investigating host–pathogen interactions during chronic mycobacterial infections.

Traditionally, *M. marinum* has been regarded as the principal causative agent of “fish tank granuloma”, an occupational disease affecting individuals exposed to contaminated aquaria, fish, or aquatic environments [13,15]. However, increasing evidence indicates that *M. chelonae* can produce clinically and histologically indistinguishable cutaneous lesions in exposed individuals [17,42]. Our findings further support this evidence and suggest that *M. chelonae* should be considered among the differential etiological agents of chronic granulomatous dermatitis associated with aquatic exposure. Rather than replacing the established concept of fish tank granuloma, these observations broaden its microbiological spectrum by emphasizing that multiple environmental mycobacterial species may produce similar clinical presentations.

The successful amplification and sequencing of the *hsp65* gene from archival FFPE tissues highlights the value of molecular diagnostics for retrospective investigations, particularly when fresh material is unavailable. The use of FFPE specimens allowed the identification of *M. chelonae* in histologically suspicious but Ziehl–Neelsen-negative lesions, demonstrating the diagnostic potential of this approach in routine pathology. Future studies integrating environmental sampling and whole-genome sequencing could provide additional insights into the epidemiology and transmission dynamics of *M. chelonae* across aquatic and human populations.

Overall, these findings highlight the importance of a comprehensive understanding of NTM ecology in environmental source reservoirs, including the distribution and persistence of clinically relevant taxa, to support epidemiological investigations, improve risk assessment, and guide strategies aimed at minimizing NTM exposure. In particular, the detection and characterization of *Mycobacterium chelonae* in aquatic environments emphasize the need for integrated surveillance approaches to improve the recognition and management of aquatic mycobacterial infections. Early identification of this opportunistic pathogen may contribute to reducing transmission within aquaculture systems and limiting potential human exposure. Therefore, coordinated veterinary, medical, and environmental surveillance within a One Health framework is essential to better understand NTM dynamics and develop effective prevention and control strategies.

## 5. Conclusions

This study identifies *Mycobacterium chelonae* as an important cause of chronic granulomatous disease in Mediterranean marine fish and confirms its involvement in human granulomatous dermatitis. The consistent absence of detectable acid-fast bacilli in Ziehl–Neelsen-stained sections highlights the limited sensitivity of conventional histochemistry and supports the routine integration of molecular assays into the diagnostic investigation of granulomatous lesions, particularly when formalin-fixed, paraffin-embedded tissues represent the only material available.

The identification of *M. chelonae* in both fish and human granulomatous lesions, together with the close genetic similarity of the detected isolates, reinforces the importance of considering this rapidly growing mycobacterium within a One Health framework. Our findings suggest that *M. chelonae* should be recognized, alongside *Mycobacterium marinum*, as a potential etiological agent of fish tank granuloma. Crucially, we report for the first time that *M. chelonae* can independently induce this condition in humans. This is evidenced by the distinct distribution of granulomatous lesions in our cohort, where positive skin biopsies were interestingly localized to the patient’s extremities. Therefore, *M. chelonae* must be included in the differential diagnosis of chronic granulomatous dermatitis in human skin samples associated with aquatic exposure.

## Figures and Tables

**Table 1:** Results of histochemistry and biomolecular analysis in fish species.

Case No	Sample ID	Species	Organ	PCR *hsp65*	Sequencing	ZN
1	I 393	Mugilidae	Brain	−		−
	I 395		Liver	−		−
	I 396		Gonad	−		−
2	I 427	Mugilidae	Liver	+	*M. chelonae*	−
3	I 1061	Mugilidae	Liver	+	*M. chelonae*	−
4	I 1072	Mugilidae	Spleen	+	*M. chelonae*	−
5	I 1085	Mugilidae	Liver	+	*M. chelonae*	−
6	I 1096	Mugilidae	Spleen	+	*M. chelonae*	−
7	I 1101	Mugilidae	Liver	−		−
8	I 1133	Mugilidae	Liver	+	*M. chelonae*	−
9	I 1197	Mugilidae	Liver	+	*M. chelonae*	−
10	I 1221	Mugilidae	Liver	−		−
11	I 1237	Mugilidae	Liver	+	*M. chelonae*	−
12	I 1253	Mugilidae	Liver	−		−
13	I 1277	Mugilidae	Liver	+	*M. chelonae*	−
14	I 1625	*S. aurata*	Liver	+	*M. chelonae*	−
15	I 1700	*S. aurata*	Spleen	+	*M. chelonae*	−
16	I 1741	*S. aurata*	Kidney	−		−
17	I 1753	*S. aurata*	Liver	+	*M. chelonae*	−
	I 1756		Spleen	−		−
18	I 1756	*S. aurata*	Spleen	−		−
19	I 1793	*S. aurata*	Liver	+	*M. chelonae*	−
	I 1794		Gonad	+	*M. chelonae*	−
20	I 2078	*S. aurata*	Heart	+	*M. chelonae*	−
21	I 2166	*S. aurata*	Heart	+	*M. chelonae*	−
22	I 2289	*S. aurata*	Heart	−		−
23	I 1450	*D. labrax*	Heart	+	*M. chelonae*	−
24	I 1462	*D. labrax*	Spleen	+	*M. chelonae*	−
25	I 1519	*D. labrax*	Kidney	+	*M. chelonae*	−
26	I 1915	*D. labrax*	Liver	+	*M. chelonae*	−

**Table 2:** Results of histochemistry and biomolecular analysis in human skin biopsies.

Case No.	Sample ID	Location	PCR *hsp65*	Sequencing	ZN
1	2279	Trunk	−		−
2	1605	Right arm	+	*M. chelonae*	−
3	1127	Back	−		−
4	684	Left leg	−		−
5	8395	Right arm	+	*M. chelonae*	−
6	8699	Right arm	+	*M. chelonae*	−
7	3019	Right hand	+	*M. chelonae*	−
8	8982	Left arm	+	*M. chelonae*	−
9	5188	Hand	−		−
10	1750	Thigh	+	*M. chelonae*	−
11	2689	Right shoulder	−		−
12	7802	Shoulder	−		−

**Table 3:** GenBank accession numbers of sequences obtained from *hsp65*-positive samples.

Sample ID	GenBank Accession Number
I427	PZ778604
I1061	PZ778605
I1072	PZ778606
I1085	PZ778607
I1096	PZ778608
I1133	PZ778609
I1197	PZ778610
I1237	PZ778611
I1277	PZ778612
I1625	PZ778613
I1700	PZ778614
I1753	PZ778615
I1793	PZ778616
I1794	PZ778617
I2078	PZ778618
I2166	PZ778619
2 (1605)	PZ778620
5 (8395)	PZ778621
6 (8699)	PZ778622
7 (3019)	PZ778623
8 (8982)	PZ778624
10 (1750)	PZ778625
I1450	PZ778626
I1462	PZ778627
I1519	PZ778628
I1915	PZ778629

## Data Availability

The nucleotide sequences generated in this study have been deposited in GenBank, and the corresponding accession numbers are reported in the Results Section (Table 3). All other datasets generated during and/or analysed during the current study are available from the corresponding author on reasonable request.
